# Preclinical evaluation of transcriptional targeting strategies for carcinoma of the breast in a tissue slice model system

**DOI:** 10.1186/bcr1353

**Published:** 2005-11-16

**Authors:** Mariam A Stoff-Khalili, Alexander Stoff, Angel A Rivera, Nilam S Banerjee, Maaike Everts, Scott Young, Gene P Siegal, Dirk F Richter, Minghui Wang, Peter Dall, J Michael Mathis, Zeng B Zhu, David T Curiel

**Affiliations:** 1 Division of Human Gene Therapy, Departments of Medicine, Surgery, Pathology and the Gene Therapy Center, University of Alabama at Birmingham, Birminham, AL 35294-2172, USA; 2 Department of Obstetrics and Gynecology, University of Duesseldorf, Medical Center, 40225 Duesseldorf, Germany; 3 Department of Plastic and Reconstructive Surgery, Dreifaltigkeits-Hospital, 50389 Wesseling, Germany; 4 Department of Biochemistry and Molecular Genetics, University of Alabama at Birmingham, Birmingham, AL 35294-2172, USA; 5 Department of Pathology, Cellular Biology, and Surgery and the Gene Therapy Center, University of Alabama at Birmingham, Birmingham, AL 35294-2172, USA; 6 Department of Cellular Biology and Anatomy, Louisiana State University Health Sciences Center, Shreveport, LA 71130, USA

## Abstract

**Introduction:**

In view of the limited success of available treatment modalities for metastatic breast cancer, alternative and complementary strategies need to be developed. Adenoviral vector mediated strategies for breast cancer gene therapy and virotherapy are a promising novel therapeutic platform for the treatment of breast cancer. However, the promiscuous tropism of adenoviruses (Ads) is a major concern. Employing tissue specific promoters (TSPs) to restrict transgene expression or viral replication is an effective way to increase specificity towards tumor tissues and to reduce adverse effects in non-target tissues such as the liver. In this regard, candidate breast cancer TSPs include promoters of the genes for the epithelial glycoprotein 2 (EGP-2), cyclooxygenase-2 (Cox-2), α-chemokine SDF-1 receptor (stromal-cell-derived factor, CXCR4), secretory leukoprotease inhibitor (SLPI) and survivin.

**Methods:**

We employed E1-deleted Ads that express the reporter gene luciferase under the control of the promoters of interest. We evaluated this class of vectors in various established breast cancer cell lines, primary breast cancer cells and finally in the most stringent preclinical available substrate system, constituted by precision cut tissue slices of human breast cancer and liver.

**Results:**

Overall, the CXCR4 promoter exhibited the highest luciferase activity in breast cancer cell lines, primary breast cancer cells and breast cancer tissue slices. Importantly, the CXCR4 promoter displayed a very low activity in human primary fibroblasts and human liver tissue slices. Interestingly, gene expression profiles correlated with the promoter activities both in breast cancer cell lines and primary breast cancer cells.

**Conclusion:**

These data suggest that the CXCR4 promoter has an ideal 'breast cancer-on/liver-off' profile, and could, therefore, be a powerful tool in Ad vector based gene therapy or virotherapy of the carcinoma of the breast.

## Introduction

Breast cancer is the most common cancer in the world. It affects 1 in 9 women in the United States where 46,000 women die from breast cancer each year despite early detection methods and advanced conventional treatments [1]. Clearly, novel therapies for breast cancer are required. Gene therapy and virotherapy constitute a novel therapeutic approach for the treatment of advanced, recurrent and metastatic breast cancer. In gene therapy approaches, a therapeutic gene for mutation compensation, immunopotentiation, or prodrug activation is transferred [2]. In virotherapy, tumor cell killing is achieved by oncolysis – virus replication induced cell killing [3]. Both of these therapeutic interventions allow for specific antitumor effects via molecular targeting strategies that exploit tumor markers.

At present, the most promising gene delivery vehicle is the recombinant adenoviral vector [4]. Whereas adenoviral vectors are understood to exhibit superior levels of *in vivo *gene transfer compared to available alternative vector systems, their present level of efficiency in clinical trials may nonetheless be suboptimal for cancer gene therapy and virotherapy applications [2]. Poor tumor cell transduction and non-specific cell infection are key factors limiting realization of the potential of breast cancer gene therapy [3,5]. Various approaches have been developed to enhance the infectivity of current vector systems to address poor cell transduction efficiency. To this end, transductional targeting strategies have attempted to re-engineer viral tropism such that target cell binding predicates specificity. In parallel, strategies have been developed to enhance the transcription selectivity of current vector systems for tumor cells by limiting ectopic expression in non-tumor cells, thus limiting treatment-associated toxicities. Transcriptional targeting strategies employ the use of a tissue specific promoter (TSP) to restrict transgene expression or viral replication to tumor cells. The ideal TSP for breast cancer would exhibit the widest differential between 'tumor on/liver off' expression profiles, which is key to ablation of liver toxicity from ectopically localized adenovirus (Ad). It is noteworthy that many promoters that exhibit specificity in plasmid based constructs do not show such specificity in Ad vectors. Thus, to achieve the specificity of viral replication (virotherapy) or of transgene expression (gene therapy) required in the context of breast cancer gene therapy, it is necessary to evaluate promoters and test them in the most stringent preclinical model available. We have, therefore, recently explored tissue slice technology via the Krumdieck Tissue Slicer [6], which offers a powerful and representative *ex vivo *model system for preclinical infectivity analysis of Ads. The human tumor tissue slice model system represents the heterogeneity of the tumor and maintains their three-dimensional structure *in vitro *[7]. Because cancer cell lines, passaged *in vitro *for years, may not reflect the biology of tumors *in vivo*, we will herein compare the specificity of TSPs of interest in cancer cell lines, primary breast cancer cells, as well as breast cancer tissue slices obtained using the Krumdieck Tissue Slicer.

In this study, we will examine epithelial glycoprotein (EGP)-2, cyclooxygenase (Cox)-2, the α-chemokine SDF-1 receptor (stromal-cell-derived-factor, CXCR4), secretory leukoprotease inhibitor (SLPI) and survivin promoters. These promoters were chosen because their corresponding genes are overexpressed in a variety of cancers, but are minimally expressed in normal host tissues.

Heretofore, these different promoters have not been systematically explored in breast cancer. Our study will thus provide valuable information for breast cancer adenoviral based gene therapy and virotherapy with respect to the most efficient transcriptional targeting strategies.

## Materials and methods

### Breast cancer cell lines and cell line culture

Breast cancer cell lines MB-468, AU-565, GI-101, MB-231 and the normal human mammary epithelial cells MCF-12A were obtained from the ATCC (American Type Culture Collection, Manassas, VA, USA). The 293 human transformed embryonal kidney cell line was purchased from Microbix (Toronto, Canada). All cell lines were maintained in a humidified 37°C atmosphere containing 5% CO_2 _and cultured with the recommended media. Infections were performed in medium with 2% v/v fetal bovine serum (Hyclone, Logan, UT, USA).

### Primary breast cancer cells and cell culture

Approval was obtained from the Institutional Review Board (University of Alabama at Birmimgham, USA) for all studies on human tissue. Primary fibroblasts were obtained from Dr NS Banerjee (Department of Biochemistry and Molecular Genetics, University of Alabama at Birmingham (UAB), Birnigham, AL, USA). Human breast cancer samples from eight patients who underwent surgery were obtained from the Department of Pathology, UAB (Table 1), and normal breast tissue was obtained from three patients who underwent mammoplasty. Breast cancer tissue was obtained following removal of the surgical specimen and confirmed to be breast cancer by a clinical pathologist. Time from harvest to cell preparation was kept at an absolute minimum (<2 h). Cells were obtained by mechanical disruption. All primary breast cancer cells were maintained in a humidified 37°C atmosphere containing 5% CO_2 _and cultured in RPMI (Ruswell Park Memorial Institute, supplemented with 10% v/v FCS, 2 mM glutamine, 100 U per ml penicillin, and 100 μg per ml streptomycin).

### Slice preparation with the Krumdieck Tissue Slicer

The Krumdieck tissue slicing system (Alabama Research and Development, Birmingham, AL, USA) was used in accordance with the manufacturer's instructions and previously published techniques [6]. An 8 mm coring device (Alabama Research and Development) was used to retrieve an 8 mm diameter core of tissue from the human breast or liver tissue sample. This was then placed in a slicer filled with ice-cold culture media. Slice thickness was set at 250 microns using a tissue slice thickness gauge (Alabama Research and Development) and slices were cut using a reciprocating blade at 30 rpm. Afterwards, these slices were stored in ice-cold culture medium that served as a wash/equlibration solution between preservation in University of Wisconsin (UW) solution (ViaSpan, Barr Laboratories Inc., Pomona, NY, USA) and culture media [7].

### Human primary breast cancer tissue slices and culture

Approval was obtained from the Institutional Review Board for all studies on human tissue. Human breast cancer samples were obtained from the same eight patients the primary breast cancer cells were obtained from (Department of Pathology, UAB; Table 1), and normal breast tissue was obtained from three patients who underwent mammoplasty. All breast cancer samples were flushed with UW solution before harvesting and kept on ice in UW solution until slicing. Time from harvest to slicing was kept at an absolute minimum (<2 h). Breast cancer tissue slices were placed into 6-well plates (1 slice/well) containing 2 ml of complete culture media (Ruswell Park Memorial Institute, supplemented with 10% v/v FCS, 2 mM glutamine, 100 U per ml penicillin, and 100 μg per ml streptomycin [7]). The plates were then incubated at 37°C/5% CO_2 _in a humidified environment. A plate rocker set at 60 rpm was used to agitate the slices for 2 h and ensure adaequate oxygenation and viability [8].

Human dermal fibroblasts were derived from adult skin by trypsinization as described [9-11]. Human dermal fibroblasts obtained from outgrowth of explant cultures were grown in Dulbecco's modified Eagle's medium (Bio Whittaker, Rockland, Maine, USA) supplemented with 10% v/v FCS, 2 mM glutamine, 100 U per ml penicillin, and 100 μg per ml streptomycin and grown as monolayers on plastic Petri dishes in the humidified atmosphere of a CO_2 _incubator at 37°C. Fibroblasts were subcultured by trypsinization and used between the third and fifth passage.

### Human primary liver tissue slices and culture

Approval was obtained from the Institutional Review Board for all studies on human tissue. Human liver samples were obtained from three seronegative donor livers from the Department of Surgery, UAB, prior to transplantation into recipients. All liver samples were flushed with UW solution before harvesting and kept on ice in UW solution until slicing. Time from harvest to slicing was kept at an absolute minimum (<2 h). Liver tissue slices were placed into 6-well plates (1 slice/well) containing 2 ml of complete culture media (Wiliam's Medium E with 2 mM glutamine, 100 U per ml penicillin, and 100 μg per ml streptomycin, 10% v/v FCS, 2 mM glutamine [7]). The plates were then incubated at 37°C/5% CO_2 _in a humidified environment. A plate rocker set at 60 rpm was used to agitate the slices for 2 h and ensure adaequate oxygenation and viability [8].

### Viruses

AdCXCR4Luc, AdSurvivinLuc, AdSLPILuc, AdEGP-2Luc, AdCox2MLuc and AdCMVLuc are replication-defective Ads with a luciferase reporter gene in the E1 region under transcriptional control of the different promoters and have been described previously [12-15]. The viruses are all isogenic and were propagated on 293 cells and purified by double CsCl density centrifugation. Physical particle concentration (viral particles/ml) was determined by OD_260 _reading, and functional virus titers (plaque-forming units/ml) were determined by plaque assay in 293 cells.

### *In vitro *gene-transfer assays of breast cancer cells

Cell lines were plated on day 1 at 30,000 cells/well on 24-well plates in 1 ml of 10% v/v GM (Growth Medium). On day 2, cells were infected with recombinant Ads at a multiplicity of infection (MOI) of 100 for 2 h in 200 μl of 2% v/v GM on a rocker. Afterwards, cells were washed once with 1 ml of PBS, and 1 ml of 10% v/v GM was added per well. Purified breast cancer primary cells were plated at 10,000 cells/well on 96-well plates in 100 μl GM on a rocker for 2 h. After 2 h, cells were infected with 100 viral particles/cell for 2 h in 20 μl of 2% v/v GM on a rocker. Afterward, cells were washed once with PBS, and 60 μl of 10% v/v GM were added per well. After 24 h, the GM was removed and cells were washed once with PBS, lysed with 200 μl (cell lines) or 20 μl (primary cells) of lysis buffer (Reporter Lysis Buffer, Promega, Madison, WI, USA) and freeze-thawed three times. These samples (20 μl) were mixed with 100 μl of luciferase assay reagent (Reporter Lysis Buffer) and measured with a Berthold (Wildbad, Germany) Lumat LB 9501. Standardization was accomplished by setting values obtained with AdCMVLuc as 100% for each cell line and primary cells of each patient.

### Viral infection of human breast cancer and human liver tissue slices

For gene transfer assays of breast cancer and liver tissue slices, all viral infections were performed with a MOI of 500 in 2% v/v FCS complete culture medium [7]. The cell number for the tissue slices was estimated at 1 × 10^6 ^cells/slice based on an approximate 10 cell slice thickness (approximately 250 μm) and 8 mm slice diameter [7]. Infections were allowed to proceed for 24 h. The medium was removed and replaced with 10% v/v FCS complete culture medium. The infected breast cancer tissue slices and human liver tissue slices were placed in cell culture lysis buffer (Promega) and homogenized with an ultra sonicator (Fisher Scientific Model 100, Pittsburgh, PA, USA) at a setting of 15 watts for 10 s. The homogenate was centrifuged to pellet the debris, and the luciferase activities were measured using the Promega luciferase assay system. Experiments were performed in triplicate. Protein concentration of the tissue homogenates was determined using a Bio-Rad DC protein assay kit (Bio-Rad, Hercules, CA, USA) to allow normalization of the gene expression data relative to the number of cells.

### Gene expression detected by real-time quantitative PCR

Total cellular RNA was extracted from 5 × 10^5 ^cells using the RNeasy Mini kit (Qiagen, Valencia, CA, USA) followed by treatment with RNase-free DNase to remove any possible contaminating DNA from the RNA samples. The fluorescent TaqMan probes and the primer pairs used for real-time PCR analysis of the five gene mRNAs encoding Cox-2, CXCR4, EGP-2, SLPI and survivin were designed using Primer Express 1.0 (Perkin-Elmer, Foster City, CA, USA) and synthesized by Applied Biosystems (Foster City, CA, USA) (Table 2). Glyceraldehyde-3-phosphate dehydrogenase (GAPDH) was used as an internal control. For the real-time PCR assay, each 9 μl PCR reaction contained 3 mM MgCl_2_, 300 μM each of dATP, dCTP, and dGTP, 600 μM dUTP, 100 nM of forward, reverse primers, and probe, 1 U of r*Tth *DNA polymerase, 0.025% BSA, and RNase-free water. A plasmid standard or 1 μl of RNA sample was added into each assay tube. Negative controls with no template were performed for each reaction series. The real-time PCR reaction was carried out using a LightCycler™ System (Roche Molecular Biochemicals, Indianapolis, IN, USA). Thermal cycling conditions were subjected to 2 minutes at 50°C, 30 minutes at 60°C, 5 minutes at 95°C, then 40 cycles of 20 s at 94°C, and 1 minute at 62°C. Data were analyzed with LightCycler software.

### Histology

Sections (4 μm) of formalin-fixed, paraffin embedded tissue slices of breast cancer tissue and normal tissue were stained with hematoxylin-and-eosin following the standard procedure and analyzed at 20× magnification with an Olympus BH2 microscope (Olympus, Tokyo, Japan). Photomicrographs were captured using a SPOT camera (Diagnostic Instruments, Sterling Heights, MI, USA) and assembled with Adobe Photoshop 6.0 (Adobe Systems, San Jose, CA, USA).

### Immunofluorescence

Presicion cut tissue slices obtained from breast cancer tissue and normal breast tissue infected with AdCMVLuc and AdCXCR4Luc were collected and frozen in isopentane chilled in liquid nitrogen. Cryosections were cut at 10 μm, and the slides were frozen at -80°C until used. The sections were air dried after removing from the freezer, fixed in 4% v/v paraformaldehyde in PBS for 10 minutes and permeabilized in PBS and 0.1% v/v Triton X-100 for 30 minutes at room temperature. Then tissue sections were incubated with 10% v/v normal rabbit serum in PBS for 30 minutes and goat anti-luciferase primary antibody (G745A, Promega) at 1:50 dilution in PBS, 10% v/v normal rabbit serum, 1% v/v BSA, and 0.1% v/v Triton X-100 overnight at 4°C. After washing three times for 3 minutes each in PBS, the sections were incubated with Alexa-488 (green fluorescence) conjugated donkey anti-goat secondary antibody (A-11055, Molecular Probes, Eugene, Oregon, USA) at 1:100 dilution in PBS for 1 h at room temperature. After being washed in PBS, the sections were mounted in 4',6-diamidino-2-phenylindole dihydrochloride (DAPI) containing fluorescence mounting medium (Vectashield, catalogue no. H-1200, Vector Laboratories Inc., Burlingame, CA, USA). Green fluorescence for luciferase protein expression was analyzed at ×20 objective magnification with an Olympus Provis AX70 fluorescence microscope using FITC and DAPI filters. Images were digitally recorded with an Axiocam charge-coupled device digital camera (Carl Zeiss, Oberkochen, Germany) and AxioVision 3.1 image capture software (Carl Zeiss). Images were finally processed using Adobe Photoshop 6.0.

### Statistics

Data are presented as mean values ± standard deviation. Statistical differences among groups were assessed with a two-tailed Student's t-test. P < 0.05 was considered significant.

## Results

### Analysis of candidate promoters for transcriptional targeting of Ad-mediated gene expression in breast cancer cell lines

**Figure 1:**
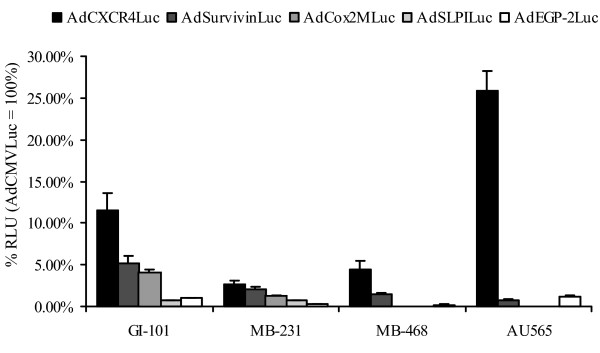
Evaluation of candidate promoters for transcriptional targeting of breast cancer cell lines. Luciferase activities in breast cancer cell lines (MB-468, AU-565, GI-101 and MB-231). These cell lines were infected with AdCXCR4Luc, AdSurvivinLuc, AdCox2MLuc, AdSLPILuc, AdEGP-2Luc and AdCMVLuc, respectively, at a multiplicity of infection of 100. Gene expression was measured 24 h after infection and is presented for the candidate promoters as percentage of cytomegalovirus promoter activity in relative light units (RLU). Each bar presents the mean of three experiments (± standard deviation).

The transcriptional activities of the CXCR4, survivin, Cox-2, SLPI and EGP-2 promoters driving luciferase expression in recombinant Ad vectors was evaluated in four human breast cancer cell lines (MB-468, AU-565, GI-101 and MB-231). Cell lines were infected with the relevant adenoviral vectors at a MOI of 10 (Fig. 1). AdCMVLuc was used to standardize for varying transduction efficiencies between cell lines, and promoter activities are therefore plotted as percentage of cytomegalovirus (CMV) driven luciferase expression [12,13,16,17]. In the breast cancer cell lines tested, each of the candidate promoters demonstrated variable relative luciferase activity between the different cell lines. The CXCR4 promoter, however, consistently showed a higher relative luciferase activity compared to the other candidate promoters. These results suggest that the CXCR4 promoter is promising for transcriptional targeting in breast cancer cell lines.

### Analysis of candidate promoters for transcriptional targeting of adenovirus-mediated gene expression in primary breast cancer cells

**Figure 2:**
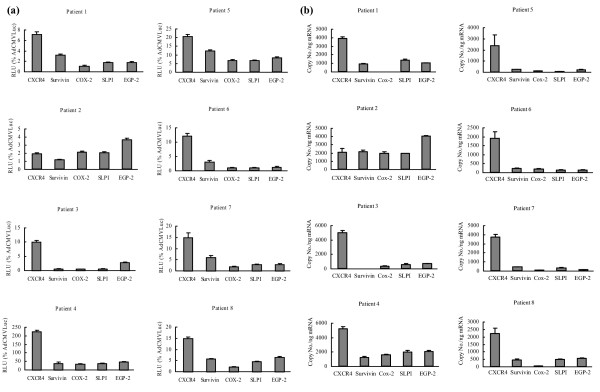
Analysis of candidate promoters for transcriptional targeting of adenovirus-mediated gene expression and correlation to the mRNA copy number of the corresponding genes in primary breast cancer cells. **(a) **Evaluation of candidate promoters for transcriptional targeting of primary breast cancer cells derived from patients. Primary breast cancer cells were isolated from eight patients and purified and infected with AdCXCR4Luc, AdSurvivinLuc, AdCox2MLuc, AdSLPILuc, AdEGP-2Luc or AdCMVLuc, at a multiplicity of infection of 100. Gene expression was measured 24 h after infection and is presented for the candidate promoters as percentage of cytomegalovirus promoter activity in relative light units (RLU). Each bar presents the mean of three experiments ± standard deviation. **(b) **Gene expression of candidate tumor-specific genes in breast cancer patient samples. Messenger RNA was extracted from human primary breast cancer samples from eight patients and reverse-transcribed into cDNA. Real-time PCR was performed to evaluate the expression of the genes encoding cyclooxygenase (Cox)-2, epithelial glycoprotein (EGP)-2, secretory leukoprotease inhibitor (SLPI), survivin and the α-chemokine SDF-1 receptor (CXCR4). The mRNA copy numbers are normalized by the glyceraldehyde-3-phosphate dehydrogenase copy number. Each bar presents the mean of three experiments ± standard deviation.

To more closely model the clinical situation, gene transfer experiments were performed using unpassaged human primary breast cancer cells. Primary cancer cells were obtained from breast cancer samples from eight patients; these patients had different stages and grades of breast cancer (Table 1). The primary breast cancer cells were infected with the relevant Ad vectors at a MOI of 10 (Fig. 2a). Again, in most of the tumor samples, the CXCR4 promoter displayed the highest luciferase activity, consistent with the results obtained in breast cancer cell lines. The exception to this trend was observed in the sample derived from patient 2, which showed low overall luciferase activity, with the highest activity in cells transduced by the Ad containing the EGP-2 promoter rather than the CXCR4 promoter. In most breast cancer samples, the relative luciferase activity of each of the promoters was significantly lower (p < 0.05) than the CMV promoter. However, in patient 4 (advanced, metastatic and recurrent breast cancer after chemo- and radiotherapy), the CXCR4 promoter activity was two-fold higher than the CMV promoter activity. These results are in line with those obtained in breast cancer cell lines, with the CXCR4 promoter showing the most promise for transcriptional targeting in breast cancer.

### Cox-2, EGP-2, SLPI, survivin and CXCR4 mRNA copy numbers correlate with the transcriptional activity of the corresponding promoters in primary breast cancer cells

To correlate transcriptional activity with the expression level of the corresponding endogenous genes in the breast cancer patient samples, Cox-2, EGP-2, SLPI, survivin and CXCR4 mRNA levels were assessed using quantitative reverse transcriptase PCR (Fig. 2b). Whereas similar levels of expression were seen for the housekeeping gene GAPDH among all the patients (data not shown), the mRNA levels of examined genes varied between the different patients. In this regard, differences in the mRNA levels of candidate genes strongly correlated with the results of the promoter evaluation. With the exception of patient 2, copy numbers for CXCR4 were significantly (p < 0.05) higher than those for EGP-2, SLPI, Cox-2 and survivin.

**Figure 3:**
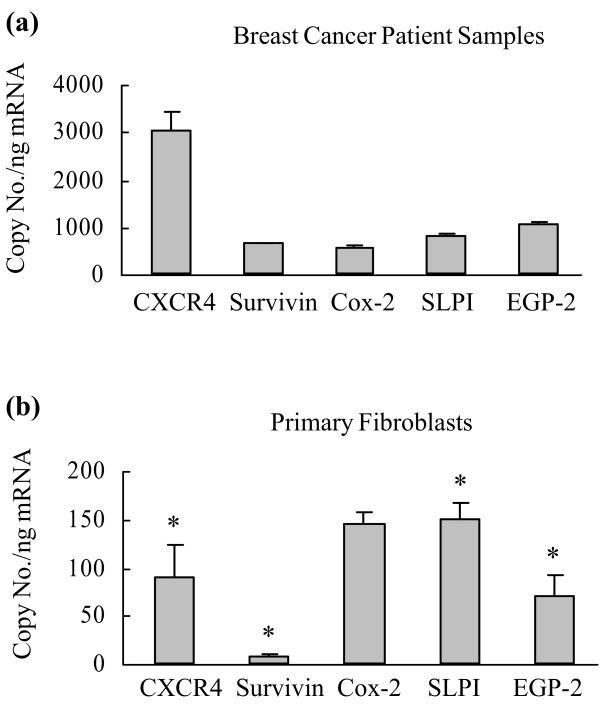
Comparison of gene expression profiles in breast cancer patient samples and normal human fibroblasts. Messenger RNA was extracted from **(a) **human primary breast cancer samples from eight patients and **(b) **human primary fibroblasts from three patients and reverse-transcribed into cDNA. Real-time PCR was performed to evaluate the expression of the genes encoding cyclooxygenase (Cox)-2, epithelial glycoprotein (EGP)-2, secretory leukoprotease inhibitor (SLPI), survivin and the α-chemokine SDF-1 receptor (CXCR4). The mRNA copy numbers are normalized by the glyceraldehyde-3-phosphate dehydrogenase copy number. Each bar presents the mean ± standard deviation. Asterisks indicate p < 0.05 for breast cancer versus normal fibroblasts.

To get an indication of the specificity of expression of these genes of interest in breast cancer tissue, we next compared the mean levels of gene expression between the eight breast cancer patient samples to levels of gene expression in three samples of individual normal primary unpassaged fibroblasts (Fig. 3; note the different scales on the ordinate). Of the candidate genes tested, mRNA levels for CXCR4 (p < 0.001), survivin (p < 0.05), SLPI (p < 0.05) and EGP-2 (p < 0.05) were significantly lower in normal primary fibroblasts compared with breast cancer tissue, while COX-2 showed no significant difference. Thus, our studies indicate that of all the tested candidate genes, the *CXCR4 *gene again demonstrates the highest potential in transcriptional targeting in breast cancer, with the highest mRNA copy number in breast cancer samples and a significantly lower copy number in normal tissue represented by primary fibroblasts.

### Analysis of candidate promoters for transcriptional targeting of adenovirus-mediated gene expression in primary breast tissue slices

**Figure 4:**
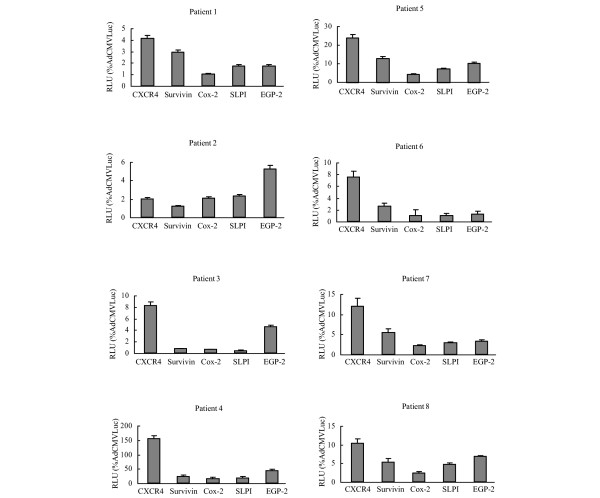
Evaluation of candidate promoters for transcriptional targeting of primary breast cancer tissue slices. Human breast cancer tissue slices were obtained from primary breast cancer samples using the Krumdieck Tissue Slicer. Slices were infected with AdCXCR4Luc, AdSurvivinLuc, AdSLPILuc, AdEGP-2Luc, AdCox2MLucor AdCMVLuc at a multiplicity of infection of 500. Gene expression was measured 24 h after infection and is presented for the candidate promoters as percentage of cytomegalovirus promoter activity in relative light units (RLU). All data points are of triplicate slices; bars represent means ± standard deviation. Cox, cyclooxygenase; CXCR4, α-chemokine SDF-1 receptor; EGP, epithelial glycoprotein; SLPI, secretory leukoprotease inhibitor.

Next, we investigated whether the results obtained with the tested candidate promoters in primary breast cancer cells could be reproduced in the most stringent available preclinical model of precision cut breast cancer tissue slices. Primary breast cancer tissue slices were obtained from the eight breast cancer patient samples (Table 1). The CXCR4 promoter consistently (with the exception of patient 2) achieved the highest luciferase activity in the breast cancer tissue slices (Fig. 4). These results are consistent with those from the breast cancer cell lines and the primary breast cancer cell model, with a strong correlation between the latter and the tissue slices for each individual patient.

### Analysis of candidate promoters for transcriptional targeting of adenovirus-mediated gene expression in normal breast tissue slices

**Figure 5:**
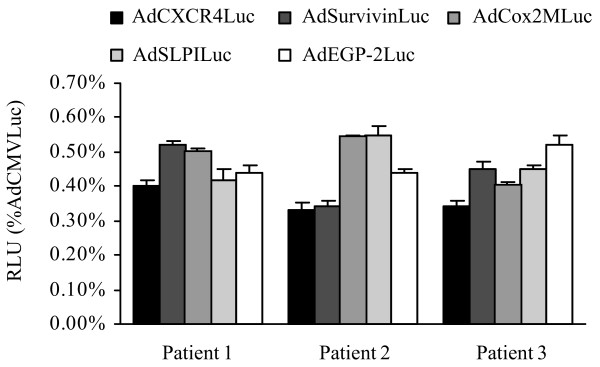
Analysis of candidate promoters for transcriptional targeting of adenovirus-mediated gene expression in normal breast tissue slices. Human normal breast tissue slices (from three patients undergoing mammoplasty) were infected with AdCXCR4Luc, AdSurvivinLuc, AdSLPILuc, AdEGP-2Luc, AdCox2MLuc or AdCMVLuc. Gene expression was measured 24 h after infection and is presented for the candidate promoters as percentage of cytomegalovirus promoter activity in relative light units (RLU). All data points are of triplicate slices; bars represent means ± standard deviation.

**Figure 6:**
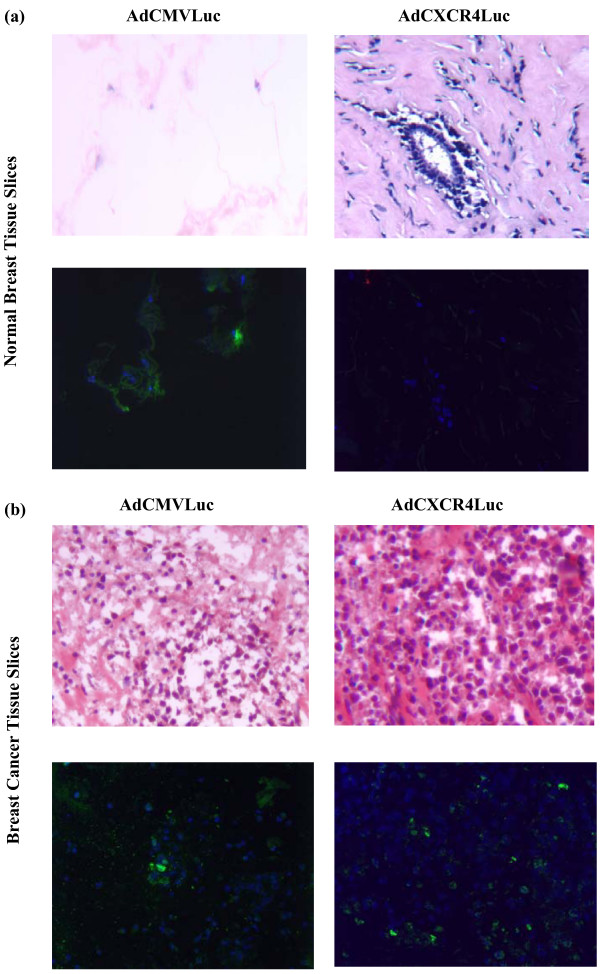
Visualization of luciferase expression pattern of AdCXCR4Luc and AdCMVLuc in normal breast tissue slices versus breast cancer tissue slices. The upper panels show the tissue morphology as revealed by hematoxylin-and-eosin staining of thin sections of **(a) **normal breast tissue slices and **(b) **breast tissue slices. Representative indirect immunfluorescent detection of luciferase expression is shown in the lower panels for **(a) **human normal breast tissue slices and **(b) **breast cancer tissue slices infected with AdCXCR4Luc and AdCMVLuc. AdCMVLuc infected **(a) **normal breast tissue slices (lower left panel) and **(b) **breast cancer tissue slices (lower left panel) show immunofluorescent detection of the luciferase protein (green fluorescence), whereas **(a) **AdCXCR4 infected normal breast tissue slices (lower right panel) do not display luciferase activity. In contrast, **(b) **AdCXCR4 infected breast cancer tissue slices reveal luciferase activity (lower right panel). DAPI (4'6-diamidino-2-phenylindole 2HCL) staining (blue) indicates nuclei. Magnification ×200.

To underline the specificity of the candidate promoters for breast cancer samples, three patient samples consisting of normal breast tissue were infected with the different transcriptionally targeted adenoviral vectors. The results indicate that the expression levels for all tested promoters were all below 0.6% relative to the CMV promoter in normal breast tissue (Fig. 5). Finally, we visualized the selective transcriptional targeting of the most promising vector tested in this study, AdCXCR4, in breast cancer tissue versus normal breast tissue (Fig. 6). In this regard, the expression pattern of the luciferase protein, the product of the luciferase gene in the recombinant Ad vector driven by the CXCR4 promoter and the CMV promoter, was determined in breast cancer tissue slices and normal breast tissue slices using immunofluorescent detection. Importantly, in normal breast tissue slices infected with AdCXCR4, no luciferase protein expression could be visualized, whereas in normal breast tissue infected with AdCMV luciferase protein, expression was seen. These data underline the previous results indicating that the CXCR4 promoter is a promising selective tool for transcriptional targeting of breast cancer.

### Evaluation of the candidate promoters in human liver tissue slices

**Figure 7:**
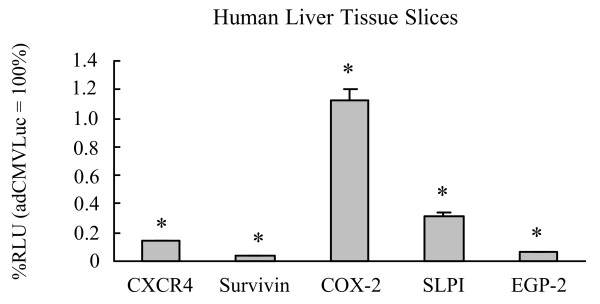
Gene expression patterns in human liver tissue slices. Human liver tissue slices (from three donors) were infected with AdCXCR4Luc, AdSurvivinLuc, AdSLPILuc, AdEGP-2Luc, AdCox2MLuc or AdCMVLuc. A luciferase assay was performed after 24 h. Gene expression was measured 24 h after infection and is presented for the candidate promoters as percentage of cytomegalovirus promoter activity in relative light units (RLU). All data points are of triplicate slices; bars represent means ± standard deviation. Asterisks indicate p < 0.05 versus AdCMVLuc. Cox, cyclooxygenase; CXCR4, α-chemokine SDF-1 receptor; EGP, epithelial glycoprotein; SLPI, secretory leukoprotease inhibitor.

A key limitation for the use of a systemic adenoviral gene therapy or virotherapy is the potential toxicity to non-target organs. Although initial specificity of the promoters was demonstrated by virtue of gene expression profiles in primary fibroblasts (Fig. 3), we next evaluated gene expression in the most relevant context, the human liver. For this, each of the candidate promoters was assessed for transgene expression in fresh-cut human liver tissue slices. In this assay, transgene expression induced by all the tested promoters (Cox-2, EGP-2, SLPI, survivin and CXCR4) was significantly less than that with the CMV promoter (Fig. 7). Of note, the survivin promoter demonstrated the lowest transgene expression of all the promoters tested in human liver tissue slices. In addition, the CXCR4 promoter displayed a significantly lower mean relative luciferase activity in the human liver tissue slices (0.18%) than in the primary breast cancer samples (37.7%) or breast cancer tissue slices (36.0%). These results demonstrate that all the tested promoters, in the context of an Ad vector, possess the key characteristic of a low level of expression in the human liver, which is considered essential for use in cancer gene therapy and virotherapy.

## Discussion

The exploitation of novel therapeutic strategies merits a high priority in the treatment of advanced, recurrent and metastatic breast cancer. Adenoviral cancer gene therapy and virotherapy have recently demonstrated promising clinical results. It has become evident, however, that one of the primary factors preventing specific and efficient gene delivery (gene therapy) or specific viral replication (virotherapy) in breast cancer is the promiscuous tropism of Ad. For the treatment of metastatic or advanced stages of breast cancer, intravenous delivery schemes of Ad vectors are desirable, but are potentially associated with ectopic localization, principally to the liver [18]. Thus, finding ways to target Ads to breast cancer cells is mandatory to reduce the risk of nonspecific gene expression and nonspecific adenoviral replication. TSPs are a promising means to genetically limit transgene expression or viral replication to tumor cells [19,20]. A wide range of promoters has been evaluated for transcriptional targeting; however, TSPs for use in breast cancer have not been systemically explored. In the present study, therefore, we compare five candidate promoters (for Cox-2, EGP-2, SLPI, survivin and CXCR4) for specific transcriptional control in breast cancer, which might hold promise in the context of future gene therapy or virotherapy regimes for this disease.

We chose the candidate promoters based upon established links to the pathobiology of cancer. In this regard, Cox-2 is an inducible isoform of the cylooxygenase family and is virtually undetectable in most tissues under physiological conditions [21]. Recently, however, overexpression of Cox-2 has been reported in colon cancers associated with familial adenomatous polyposis, as well as sporadic colorectal cancer, and in cancers of the ovary, stomach, lung, esophagus, liver, pancreas and skin [15,22-26]. Approximately 50% of human breast tumors have been reported to express Cox-2 [27,28]. SLPI is a 12 kDa serine protease inhibitor expressed in some human carcinomas, including breast, ovary, lung and endometrium [14,29]. Survivin is a member of the IAP (inhibitor of apoptosis protein) protein family, members of which have roles in the growth and progression of a variety of cancers. Recently, the gene encoding survivin has been described as being selectively expressed in some of the most common human neoplasms, such as breast cancer [30], pancreatic cancer [31], esophageal carcinoma [32], primary glioblastoma [33], ovarian cancer [34], and melanomas [35], but is undetectable in normally differentiated tissues [31]. Recently, EGP-2, also referred to as 17-1A or EpCAM, has been shown to be expressed as a stable transmembrane protein at high levels on a variety of epithelial tissue derived cancers, such as those of the breast, pancreas, gonads, gastrointestinal, respiratory and urinary tracts [36]. The function of the transmembrane glycoprotein EGP-2 is still not well understood, although recent reports have suggested it has a role as a modulator of invasiveness and metastasis [37]. CXCR4, identified as a co-receptor for HIV-1, is a chemokine receptor recently implicated in the metastatic homing of breast cancer cells to alternative tissues [30]. It has been reported that *CXCR4 *gene expression is markedly up-regulated in breast cancer cells, but is undetectable in normal mammary primary epithelial and stromal cells [38]. Furthermore, recent evidence points to the SDF-1α-CXCR4 complex as having a role in progression to metastasis in several tumor contexts [39,40].

As demonstrated in our study, the CXCR4 promoter showed the highest level of expression and specificity for breast cancer tissue, corroborating the evidence in the literature for a clear link to breast cancer pathobiology. Of note, although the absolute level of expression was less compared to the CMV promoter in the different patients (exception patient 4), it is mainly the level of specificity for a particular tissue type that will determine successful application in a clinical setting. Recent studies evaluating promoter activity in the context of ovarian cancer gene therapy have demonstrated that even promoter activities lower than 5% can be regarded sufficient to achieve therapeutic efficacy [41]. Unlike previous studies [7,27,28] the gene encoding Cox-2 was not significantly over-expressed in our breast cancer patient samples; however, variable gene expression may result from different patient tumor samples.

Because human trials have suggested that established cell lines may exhibit distinct properties different from original breast cancer cells, likely due to the culturing *in vitro*, we employed primary cultures to more closely resemble breast cancer cell phenotypes in patients [42]. Primary cancer cells were obtained by dissociation of epithelial tissue followed by culturing of the cells. As mechanical and enzymatic cell dispersion will result in loss of polarity, changes in protein expression patterns and loss of tissue structure, results obtained in these primary cells might not reflect the *in vivo *situation with respect to adenoviral infection [42]. This highlights the need to carefully evaluate the optimal Ad vector in the most stringent available preclinical model. For this reason, we have recently introduced a novel *ex vivo *tissue slice model employing the Krumdieck Tissue Slicer, which offers a powerful and representative system for preclinical infectivity analysis of tumor samples [7]. Similar findings in two different culture systems, one being organotypic and the other being a primary monolayer cell culture, validate our results. Futhermore, our results demonstrate a strong correlation between the promoter activity and expression levels of the corresponding genes in primary breast cancer samples. This correlation indicates that these results could be used to tailor transcriptionally targeted vectors to individual patient tumors based on determination of the endogenous level of the promoter activity in breast cancer samples.

For adenoviral gene therapy, ectopic transduction of non-tumor target cells can elicit vector-associated toxicities, such that hepatotropism is a major concern [43-45]. All of the promoters in the present study have previously been shown to have a 'liver off' status using mouse liver. We endeavored to test the function of these promoters in the Krumdieck Tissue Slice system, which closely reproduces the patient's situation using human liver tissue slices. The human liver tissue slice model has been shown previously to provide a valid means for preclinical assay of potential Ad-based hepatotoxicity [7,46]. Importantly, the activity of all of the promoters used in this study was low in human liver tissue slices.

## Conclusion

This is the first time that precision cut tissue slice technology has been applied in breast cancer. For this reason, we endeavored to test the five candidate promoters in precision cut tissue slices of patient breast cancer samples. Clearly, the activity of the CXCR4 promoter was superior in established breast cancer cell lines, primary breast cancer cells, and primary breast cancer tissue slices, whereas the CXCR4 promoter activity was low in normal breast tissue and, most importantly, human liver. The *CXCR4 *gene plays a major role in progression and metastasis of various tumor types and is, therefore, a rational target for breast cancer therapy. This promoter may thus be a promising candidate for tumor-specific gene therapy and virotherapy applications in breast cancer. The important value of this study is that we systemically explored TSPs for breast cancer in the most stringent available preclinical model. These data using comparative tumor and non-tumor liver tissue slices may establish the foundation for rational development of selective transcriptionally Ad-based gene therapy and virotherapy.

## Abbreviations

Ad = adenovirus; BSA = bovine serum albumin; CAR Coxsackie-Adenovirus-Receptor; CMV = cytomegalovirus; Cox = cyclooxygenase; CXCR4 = α-chemokine SDF-1 receptor; DAPI = 4',6-diamidino-2-phenylindole dihydrochloride; EGP = epithelial glycoprotein; FCS = fetal calf serum; GAPDH = glyceraldehyde-3-phosphate dehydrogenase; MOI = multiplicity of infection; PBS = phosphate-buffered saline; PCR = polymerase chain reaction; SLPI = secretory leukoprotease inhibitor; TSP = tissue specific promoter; UAB = University of Alabama at Birmingham; UW = University of Wisconsin. 

## Competing interests

The authors declare that they have no competing interests.

## Authors' contributions

All authors listed contributed to the production of this manuscript. AS, AR and MSK amplified the Ads. AR performed the Ad gene transfer to breast cancer cell lines, AS to the primary breast cancer cells and primary fibroblasts, MSK to the breast cancer tissue slices and human liver tissue slices. MSK carried out the precision cut technique with the breast cancer tissue samples, AS with the human liver tissue samples. MW performed the real-time PCR. NSB performed the histology and the immunofluoresecence experiments. GPS and SY provided the breast cancer samples and the patient data. ZZ made substantial contributions to the conception and design of the study. MM and ME have been involved in revising the manuscript critically for important intellectual content. DTC, DFR and PD gave final approval of the version to be published. MSK performed statistical analysis and wrote the manuscript.

## Figures and Tables

**Table 1:** Stages and grades of breast cancer samples from eight patients

Patient	Age	Sex	Race	Stage	Grade	Size (cm)	Histological type	Laterality	Adjuvant therapy	Previous radiation	ER	PR	Erb2
1	46	F	AA	pT1pN1pMx	3	1.8	Infiltrating mammary	R	No	No	-	-	+
2	42	F	W	pT2pN0pMx	3	2.2	Ductal	L	No	No	+	+	-
3	63	F	W	pT1cpN1pMx	3	2.0	Ductal, DCIS, LCIS	L	No	No	+	+	++
4	53	F	AA	pT3pN1pMx	?	5.1	Ductal	R (1998)	No	No	+	-	-
								R (2000)	Adriamycin, Cytoxan, Taxol, Tamoxifen	Yes	+	+	-
								R (2004)	Femara, Taxotere	No	-	-	-
							Metastases to axilla	L (2005)	?	?	-	+	++
5	47	F	W	pT2pN1pMx	2	2.5	Ductal	R	No	No	-	-	+
6	42	F	AA	pT2pN1pMx	2	2.1	Ductal	R	No	No	-	-	-
7	55	F	W	pT1pN0pMx	1	1.5	Ductal	L	No	No	+	+	-
8	64	F	W	pT1pN1pMx	3	1.9	Ductal	R	No	No	-	-	+

DCIS, ductal carcinoma *in situ*; F, female; L, left; LCIS, lobular carcinoma *in situ*; R, right; W, white; AA; African-american, ER, estrogen receptor, P, progesterone receptor.

**Table 2:** List of oligo pairs and probes used in quantitative RT-PCR

Gene	Accession*	Forward primer	Reverse primer	Probe
Cox-2	M90100	5'-TGATCCCCAGGGCTCAAAC-3'	5'-AGCTGGCCCTCGCTTATGA-3'	6FAMCTTTGCCCAGCACTTCACGCATCAGTAMRA
CXCR4	AF348491	5'-AACCAGCGGTTACCATGGAG-3'	5'-CTTCATGGAGTCATAGTCCC CTG-3'	6FAMACACTTCAGATAACTACACCGAGGAAATGGGCTAMRA
EGP-2	NM_002354	5'-GGGCCCTCCAGAACAATGA-3'	5'-TGCACTGCTTGGCCTTAAAGA-3'	6FAMCCGCTCTCATCGCAGTCAGGATCATTAMRA
Survivin	U75285	5'-TGGAAGGCTGGGAGCCA-3'	5'-GAAAGCGCAACCGGACG-3'	6FAMTGACGACCCCATAGAGGAACATAAAAAGCATTAMRA
SLPI	NM_003064	5'-TCCTGACACTTGTGGCATCAA-3'	5'-GGCTTCCTCCTTGTTGGGTT-3'	6FAMTGCCTGGATCCTGTTGACACCCCTAMRA

Cox, cyclooxygenase; CXCR4, α-chemokine SDF-1 receptor; EGP, epithelial glycoprotein; SLPI, secretory leukoprotease inhibitor.

* Gene Bank Accession Number
